# The Infection Rate of COVID-19 in Wuhan, China: Combined Analysis of Population Samples

**DOI:** 10.2196/20914

**Published:** 2020-08-14

**Authors:** Hui-Qi Qu, Zhangkai Jason Cheng, Zhifeng Duan, Lifeng Tian, Hakon Hakonarson

**Affiliations:** 1 Center for Applied Genomics The Children’s Hospital of Philadelphia Philadelphia, PA United States; 2 Department of Radiation Oncology Sun Yat-sen University Cancer Center Guangzhou China; 3 Institute of Allergy and Immunology School of Medicine Shenzhen University Shenzhen China; 4 Divisions of Human Genetics and Pulmonary Medicine The Perelman School of Medicine University of Pennsylvania Philadelphia, PA United States

**Keywords:** infectious disease, COVID-19, infection rate, China, Wuhan, fatality, public health, diagnosis

## Abstract

**Background:**

The coronavirus disease (COVID-19) pandemic began in Wuhan, China, in December 2019. Wuhan had a much higher mortality rate than the rest of China. However, a large number of asymptomatic infections in Wuhan may have never been diagnosed, contributing to an overestimated mortality rate.

**Objective:**

This study aims to obtain an accurate estimate of infections in Wuhan using internet data.

**Methods:**

In this study, we performed a combined analysis of the infection rate among evacuated foreign citizens to estimate the infection rate in Wuhan in late January and early February.

**Results:**

Based on our analysis, the combined infection rate of the foreign evacuees was 0.013 (95% CI 0.008-0.022). Therefore, we estimate the number of infected people in Wuhan to be 143,000 (range 88,000-242,000), which is significantly higher than previous estimates. Our study indicates that a large number of infections in Wuhan were not diagnosed, which has resulted in an overestimated case fatality rate.

**Conclusions:**

Increased awareness of the original infection rate of Wuhan is critical for proper public health measures at all levels, as well as to eliminate panic caused by overestimated mortality rates that may bias health policy actions by the authorities.

## Introduction

In December 2019, the first cases of coronavirus disease (COVID-19) were reported in Wuhan, China, a megacity with a population of approximately 11 million people. To prevent the spread of this highly infectious disease, the government initiated a city-wide lockdown on January 23, 2020. However, despite these efforts, COVID-19 spread to many countries across the world, reaching pandemic levels, and continues to be a serious public health concern due to its high mortality rate. According to the large-sample analysis by Wu and McGoogan [1], China’s case fatality rate (CFR) was 2.3%—that is, 1023 deaths from 44,672 confirmed cases as of February 11, 2020, with a significant proportion of cases originating from Wuhan. The large number of infected people in Wuhan put a tight strain on essential medical resources. The city had a much higher mortality rate (according to Feb 10th statistics: CFR=4.05% [748 deaths/18,454 diagnoses]; Apr 24th statistics: CFR=7.69% [3869 deaths/50,333 diagnoses]) than the rest of China. The overall CFR of 2.3% for China was likely overestimated, due to strained medical resources and a large number of undiagnosed patients. According to a recent study, 78% of those who had been infected were asymptomatic [2]. Therefore, a large number of asymptomatic infections in Wuhan might have never been diagnosed, which contributed to the overestimated CFR. An accurate estimation of the infection rate is therefore important to assess Wuhan’s CFR precisely.

## Methods

Using Markov Chain Monte Carlo methods, Wu et al [3] estimated that 75,815 individuals (95% CI 37,304-130,330) had been infected in Wuhan as of January 25, 2020. Following this, a number of foreign governments evacuated their citizens and performed thorough etiological tests on them. This group of evacuees can serve as a “random” sample to estimate the infection rate in Wuhan. With internet search as an important source of epidemiologic information on COVID-19 [4], we performed a combined analysis of the infection rates of these population samples using publicly available data (Table 1), instead of a simple pooled calculation, considering potential differences in lifestyles and pathogen exposure across different populations. The combined analysis was done using the Comprehensive Meta-Analysis Software (Biostat, Inc).

**Table 1:** Number of infected people from different countries.

Country	Evacuation date	Confirmed cases (n=14), n	Evacuees (n=1401), n
Japan [5]	N/A^a^	9	566
Korea [6-8]	January 31, 2020	1	368
Germany [9]	February 1, 2020	2	124
Singapore [10-12]	January 30, 2020	1	92
Italy [13]	February 2, 2020	1	56
United States [14]	January 29, 2020	0	195

^a^N/A: not applicable.

## Results

**Figure 1:**
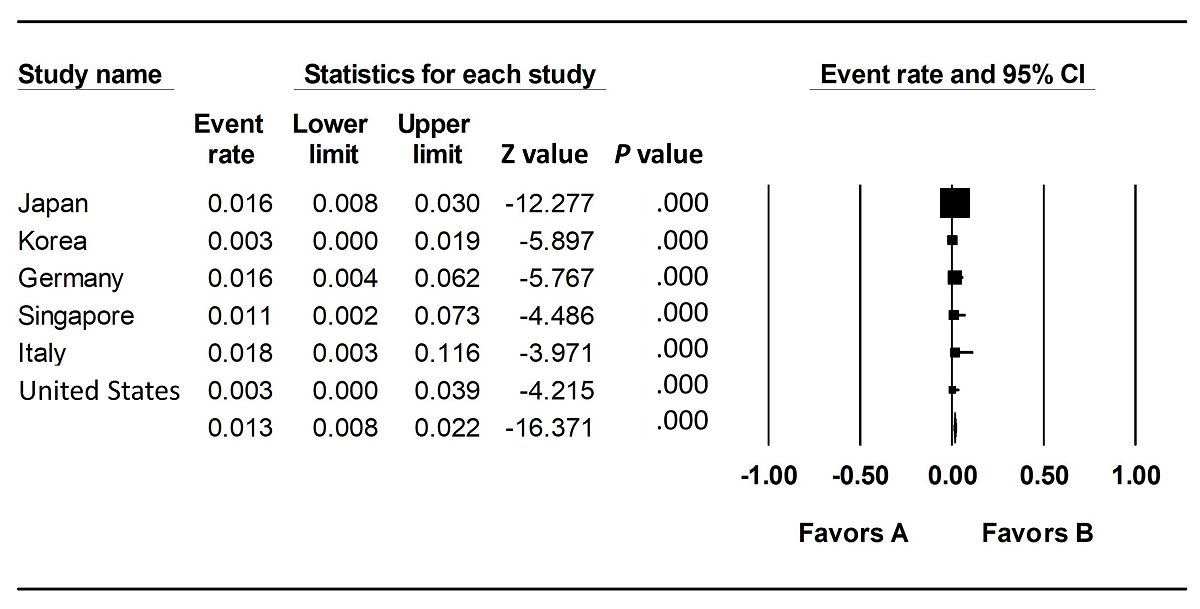
Combined analysis of infection rates of different populations.

Our analysis demonstrates that there is no significant heterogeneity across different population samples (heterogeneity test *P*=.491). The combined infection rate is 0.013 (95% CI 0.008-0.022) (Figure 1). Based on our results, we estimate the number of infected people in Wuhan, China, to be 143,000 (range 88,000-242,000), which is significantly higher than the estimate proposed by Wu et al [3].

## Discussion

Our estimate indicates that a large number of infections in Wuhan were not diagnosed. The number of undiagnosed cases in late January and early February is larger than the final diagnosed count reported to date (n=50,333), which has resulted in an overestimated CFR. In addition, our study suggests that the lower CFR (0.51%) estimated by the Centre for Evidence-Based Medicine [15] does not indicate viral variants and loss of virulence. Taken together, increased awareness of the original infection rates in Wuhan, China, is critically important for appropriate public health measures at all levels, as well as to eliminate panic caused by overestimated mortality rates that may bias health policy actions by the authorities.

## Abbreviations

COVID-19: coronavirus disease
CFR: case fatality rate
